# Bergamot Reduces Plasma Lipids, Atherogenic Small Dense LDL, and Subclinical Atherosclerosis in Subjects with Moderate Hypercholesterolemia: A 6 Months Prospective Study

**DOI:** 10.3389/fphar.2015.00299

**Published:** 2016-01-06

**Authors:** Peter P. Toth, Angelo M. Patti, Dragana Nikolic, Rosaria V. Giglio, Giuseppa Castellino, Teresa Biancucci, Fabiana Geraci, Sabrina David, Giuseppe Montalto, Ali Rizvi, Manfredi Rizzo

**Affiliations:** ^1^CGH Medical Center, SterlingIL, USA; ^2^School of Medicine, University of Illinois, PeoriaIL, USA; ^3^Johns Hopkins University School of Medicine, BaltimoreMD, USA; ^4^Biomedical Department of Internal Medicine and Medical Specialties, University of PalermoPalermo, Italy; ^5^Euro-Mediterranean Institute of Science and TechnologyPalermo, Italy; ^6^Department of Biological, Chemical and Pharmaceutical Sciences and Technologies, University of PalermoPalermo, Italy; ^7^Department of Experimental Biomedicine and Clinical Neuroscience, University of PalermoPalermo, Italy; ^8^Consiglio Nazionale delle Ricerche, Istituto di Biomedicina e Immunologia Molecolare “Alberto Monroy”Palermo, Italy; ^9^Division of Endocrinology, Diabetes and Metabolism, University of South Carolina School of Medicine, ColumbiaSC, USA

**Keywords:** Bergamot, cardiovascular risk, carotid IMT, hypercholesterolemia, LDL subclasses

## Abstract

**Background:** Some patients experience statin-induced side effects or prefer nutraceutical approaches for the treatment of dyslipidemia. This has led to a search for alternative therapeutic approaches for dyslipidemia management. In recent studies *Citrus bergamia* (known as Bergamot) juice was able to reduce serum levels of lipids. Such benefit may be attributed to high amounts of flavonoids contained in Bergamot fruit juice (neoeriocitrin, neohesperidin, naringin). The aim of the present study was to fully investigate the effects of a Bergamot extract on cardio-metabolic parameters, including plasma lipids, atherogenic lipoproteins and subclinical atherosclerosis.

**Methods:** Eighty subjects (42 men and 38 women, mean age: 55 ± 13 years) with moderate hypercholesterolemia [e.g., with plasma LDL-cholesterol concentrations between 160 and 190 mg/dl (between 4.1 and 4.9 mmol/l)] were included. A Bergamot-derived extract (Bergavit R^®^) was given at a fixed dose daily (150 mg of flavonoids, with 16% of neoeriocitrin, 47% of neohesperidin and 37% of naringin) for 6 months. Lipoprotein subfractions were assessed by gel electrophoresis. With this methodology low density lipoprotein (LDL) subclasses are distributed as seven bands (LDL-1 and -2 as large LDL, and LDL-3 to -7 as atherogenic small, dense LDL). Subclinical atherosclerosis was assessed by carotid intima-media thickness (cIMT) using B-mode ultrasound.

**Results:** After 6 months, Bergavit R^®^ reduced total cholesterol (from 6.6 ± 0.4 to 5.8 ± 1.1 mmol/l, p < 0.0001), triglycerides (from 1.8 ± 0.6 to 1.5 ± 0.9 mmol/l, p = 0.0020), and LDL-cholesterol (from 4.6 ± 0.2 to 3.7 ± 1.0 mmol/l, p < 0.0001), while HDL- cholesterol increased (from 1.3 ± 0.2 to 1.4 ± 0.4 mmol/l, p < 0.0007). In addition, a significant increase in LDL-1 (from 41.2 ± 0.2 to 49.6 ± 0.2%, p < 0.0001) was accompanied by decreased small, dense LDL-3, -4, and 5 particles (from 14.5 ± 0.1 to 9.0 ± 0.1% p < 0.0001; 3.2 ± 0.1 to 1.5 ± 0.1% p = 0.0053; 0.3 ± 0.0% to 0.1 ± 0.0% p = 0.0133, respectively). cIMT also decreased from 1.2 ± 0.4 to 0.9 ± 0.1 mm (p < 0.0001).

**Conclusion:** This is the first study investigating the effects of Bergamot flavonoids supplementation on cardio-metabolic risk in dyslipidemic subjects. Bergavit R^®^ (Bergamot juice extract) supplementation significantly reduced plasma lipids and improved the lipoprotein profile. cIMT was also reduced significantly over a relatively short time frame of 6 months.

## Introduction

Statins are the most commonly used class of drugs [3-hydroxy-3-methylglutaryl Co-enzyme A (HMG-CoA) reductase inhibitors] to lower serum cholesterol levels, with favorable effects on both plasma lipids and lipoproteins (Rizzo and Berneis, 2006a,b; Rizzo et al., 2007; Nikolic et al., 2013; Garcia-Rios et al., 2014). To this regard, particular attention has been focused on low density lipoprotein cholesterol (LDL-C) since several lines of evidences suggest that they are directly and independently associated to cardiovascular (CV) risk (Adhyaru and Jacobson, 2015). However, despite several advances in the pharmacological strategies for maintaining lipid homeostasis, some treated patients do not reach their LDL-C goal and remain at increased CV risk (Alagona and Ahmad, 2015), while others experience intolerance especially at high doses of statins (i.e., myopathy, rhabdomyolysis, hepatotoxicity, growth retardation in pediatric population; Banach et al., 2015a,b). In such situations certain alternative therapeutic approaches including the use of dietary supplements and nutraceuticals may be prudent (Patti et al., 2015).

A number of supplements for dyslipidemia are available in the market with beneficial effects on plasma lipids, although their impact on CV risk remains largely unknown (Mannarino et al., 2014). In this context, several studies have demonstrated multiple health-related properties of the *Citrus* flavonoids on CV protection (Benavente-Garcia and Castillo, 2008). Bergamot is the common name of the fruit *Citrus bergamia Risso* (family *Rutaceae*) which differs from other *Citrus* fruits in the composition and content of several distinct flavonoids, such as neoeriocitrin, neohesperidin, naringin (Dugo et al., 2005; Nogata et al., 2006). Preclinical and clinical studies indicated a hypo-cholesterolemic property of *C. bergamia* flavonoids (Trombetta et al., 2010; Mollace et al., 2011; Sakurada et al., 2011; Graziano et al., 2012; Di Donna et al., 2014; Risitano et al., 2014). However, the effects of Bergamot flavonoids on lipoprotein sizes and subclasses are still largely unknown. A Bergamot juice derived flavonoid extract, Bergavit^®^ (Bionap, Italy) contains about 28–30 % of flavonoids (including naringin, neoeriocitrin, and neohesperidin).

The aim of the present study was to elucidate the effects of Bergavit^®^ supplementation on cardio-metabolic parameters, including plasma lipids, atherogenic lipoproteins and subclinical atherosclerosis in a 6-month prospective clinical intervention study.

## Materials and Methods

### Patients and Methods

A total of 80 subjects (42 men and 38 women, mean age: 55 ± 13 years) with moderate hypercholesterolemia [e.g., with plasma LDL-C concentrations between 160 and 190 mg/dl (between 4.1 and 4.9 mmol/l)] were included in the present study. All subjects were referred to our Unit of Diabetes and CV Prevention for a clinical evaluation and were naïve to statin treatment. The study design included a medical examination, anthropometric data collection, biochemical analyses, and eco-color-Doppler examination of carotid arteries. The procedures used were in agreement with the Helsinki Declaration of 1975 as revised in 1983, and were approved by the Ethics Council of the University of Palermo, Italy. The study is registered in clinicaltrials.gov (NCT02205567).

**FIGURE 1:**
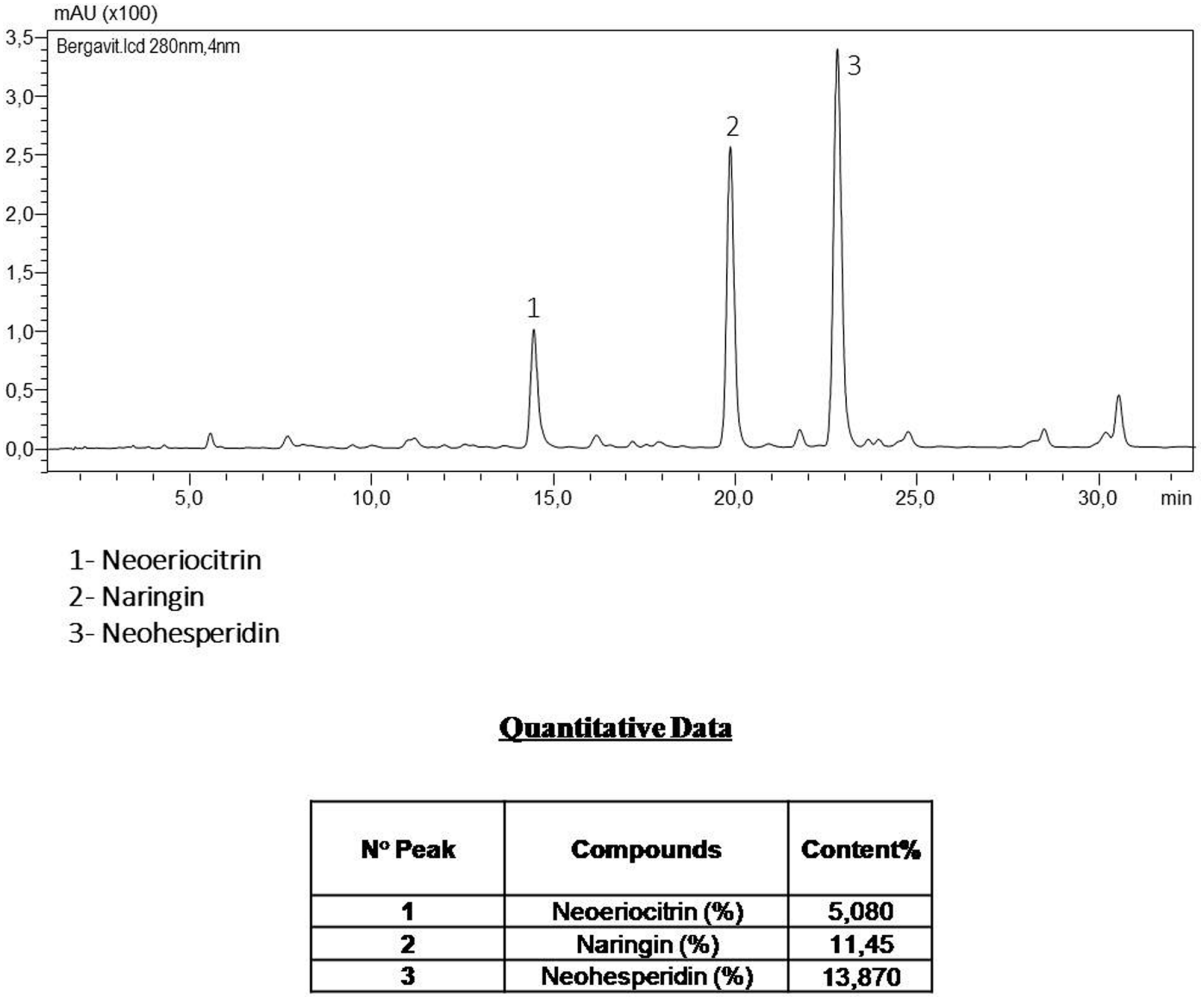
**The components of Bergavit^®^ separated by high-performance liquid chromatography (HPLC-DAD Analysis 280 nm), as determined by Bionap.** The chromatographic analyses are determined on a Shimadzu LC system by Bionap.

All subjects gave informed consent before entering the study. At admission they underwent a medical examination and were excluded from the study if they had clinical evidence of severe hepatic or renal diseases. All subjects received daily Bergamot derived flavonoid extract, Bergavit^®^ (Bionap, Italy), containing 150 mg of flavonoids, with 16% of neoeriocitrin, 47% of neohesperidin, and 37% of naringin (as determined by Bionap, see **Figure 1**), for 6 months. All medical and biochemical procedures were performed at baseline and after 6 months of supplementation.

### Analytical Method: Determination of Flavanones in Bergavit^®^

#### Instrumentation and Analytical Conditions

The analyses were carried out by Bionap (Catania, Italy) on a Shimadzu LC system, coupled to an SPD-M20A photodiode array detector (UV-DAD). The chromatographic separation was performed, also by Bionap (Catania, Italy), on a Kinetex C18 column (150 mm × 4.6 mm id-5 μm ip; Phenomenex) thermostated at 20°C; the mobile phases used were the following: Water (HPLC grade)/0.1% Formic Acid (eluent A) and Acetonitrile (HPLC grade)/0.1% Formic Acid (eluent B) (Sigma–Aldrich, Milan, Italy) using the following elution gradient: 0–5 min 13% B; 5–35 min 25% B; 35–38 min 90% B; 38–43 min 90% B; 43–45 min 13% B; 45–50 min 13% B. The flow was 1.0 mL/min and the injection volume was 5 μL (autosampler). The UV-Vis spectra were acquired on the range 190–800 nm, while the chromatograms were extracted at 280 nm, the aaa_max_ for flavanones compounds. The quantification of flavanones compounds was carried out making the calibration curves for neoeriocitrin, naringin, and noehesperidin. Sample was dissolved in water and filtered under 0.45 μm membrane filter prior HPLC injection.

### Biochemical Analyses

At baseline and after 6 months of Bergavit^®^ supplementation serum samples were collected after a 14 h overnight fast and stored for subsequent analysis. Total cholesterol (TC), triglycerides (TG), and high density lipoprotein cholesterol (HDL-C) were measured by routine laboratory methods, while LDL-C was calculated using the Friedewald formula.

We assessed a total of 11 distinct lipoprotein subclasses including very low-density lipoproteins (VLDL), 3 intermediate density-lipoprotein (IDL A, IDL B and IDL C) and 7 LDL subfractions. LDL subclasses were assessed by non-denaturing, linear polyacrylamide gel electrophoresis (Lipoprint, Quantimetrix Corporation, USA), in Palermo, Italy, as previously reported (Berneis et al., 2010; Scichilone et al., 2013; Rizzo et al., 2014). This is the only Food and Drug Administration-approved diagnostic tool for lipoprotein subfraction testing (Mikhailidis et al., 2011a). Briefly, the procedure was performed for 60 min with 3 mA for each gel tube. Each electrophoresis chamber involved two quality controls. The LDL bands in the sample were identified according to their mobility using VLDL as the starting reference point and HDL as the leading reference point. The electrophoresed gels were scanned using a digital scanner and a Mac computer (Apple Computer Inc, USA). The relative area for each lipoprotein band was determined and multiplied by the TC concentration of the sample and expressed in mg/dl. LDL subclasses were distributed as seven bands (LDL1 to LDL7, respectively; Berneis et al., 2010; Scichilone et al., 2013; Rizzo et al., 2014). LDL1 and -2 are defined as large LDL, while LDL3 to -7 as small, dense LDL (Mikhailidis et al., 2011a).

### Color Doppler Ultrasound of Carotid Arteries

B-mode real-time ultrasound was performed at baseline and after 6 months of therapy to evaluate the arterial wall thickness in the carotid arteries. All examinations were performed in Palermo, Italy, by a single examiner (A.M.P.) using a single sonographer (Medison SonoAce Pico, with a probe of 7.5–10.0 MHz) in a blinded manner; the examiner did not have access to previous scans. The ultrasound examination was performed in a standardized manner with fixed angles of insonation.

As previously reported (Corrado et al., 2006), subjects were examined in the supine position, and each carotid wall or segment was examined to identify the thickest intimal-medial site. Each scan of the common carotid artery began just above the clavicle, and the transducer was moved to the carotid bifurcation and along the internal carotid artery. Three segments were identified and measured in anterior and posterior planes on each side: the distal 1.0 cm of the common carotid artery proximal to the bifurcation, the bifurcation itself, and the proximal 1.0 cm of the internal carotid artery. At each of these sites we determined the carotid intima-media thickness (cIMT), defined as the distance between the echogenic line representing the intimal blood interface and the outer echogenic line representing the adventitial junction. The maximum cIMT value was used for analysis and determined as the mean of the maximum cIMT of near- and far-wall measurements of both the left and right side arteries for each of the three arterial segments.

### Statistical Analysis

Statistical analysis was performed using SPSS software (V.17.0 for Windows, SPSS Inc., Chicago, IL, USA). Univariate analysis was performed using paired *t*-test. We used ANOVA to test the relationships between the therapeutical changes in LDL-C and baseline plasma LDL-C, expressed in quartiles. We calculated the quartiles as following: the first quartile (Q1) includes subjects with the lowest LDL-C baseline levels (between the lowest value and top 25%), the second quartile (Q2) includes subjects with baseline levels between top 25% and top 50%, the third quartile (Q3) includes subjects with baseline levels between top 50% and top 75%, and the forth quartile (Q4) includes subjects with the highest LDL-C baseline levels (between top 75% and the highest value). Correlation analysis was performed using the Spearman rank correlation method.

## Results

Baseline characteristics of all subjects are shown in **Table 1**. None of them had to discontinue the supplementation and no adverse events were recorded.

**Table 1:** Baseline characteristics of all subjects (*n* = 80).

Age (years)	55 ± 13
Women, n (%)	38 (48)
Dyslipidemia duration (years)	5 ± 8
Smoking habit, n (%)	20 (25)
Family history of cardiovascular disease, n (%)	51 (64)
Diabetes, n (%)	42 (53)
Hypertension, n (%)	48 (60)
Obesity, n (%)	28 (35)


**FIGURE 2:**
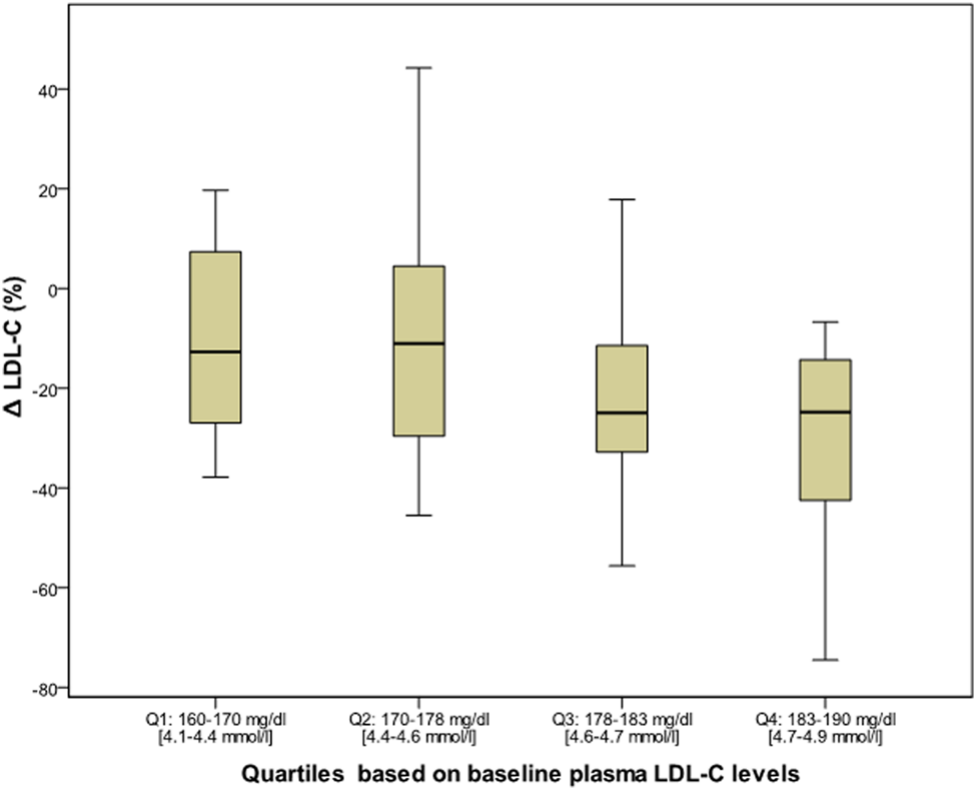
**Box plots of Δ LDL-C (%) based on quartiles of baseline plasma LDL-C levels in all subjects (*n* = 80) after 6 months of Bergavit^®^ (150 mg of flavonoids, with 16% of neoeriocitrin, 47% of neohesperidin, and 37% of naringin, daily) supplementation.** LDL-C, low-density lipoprotein cholesterol.

**FIGURE 3:**
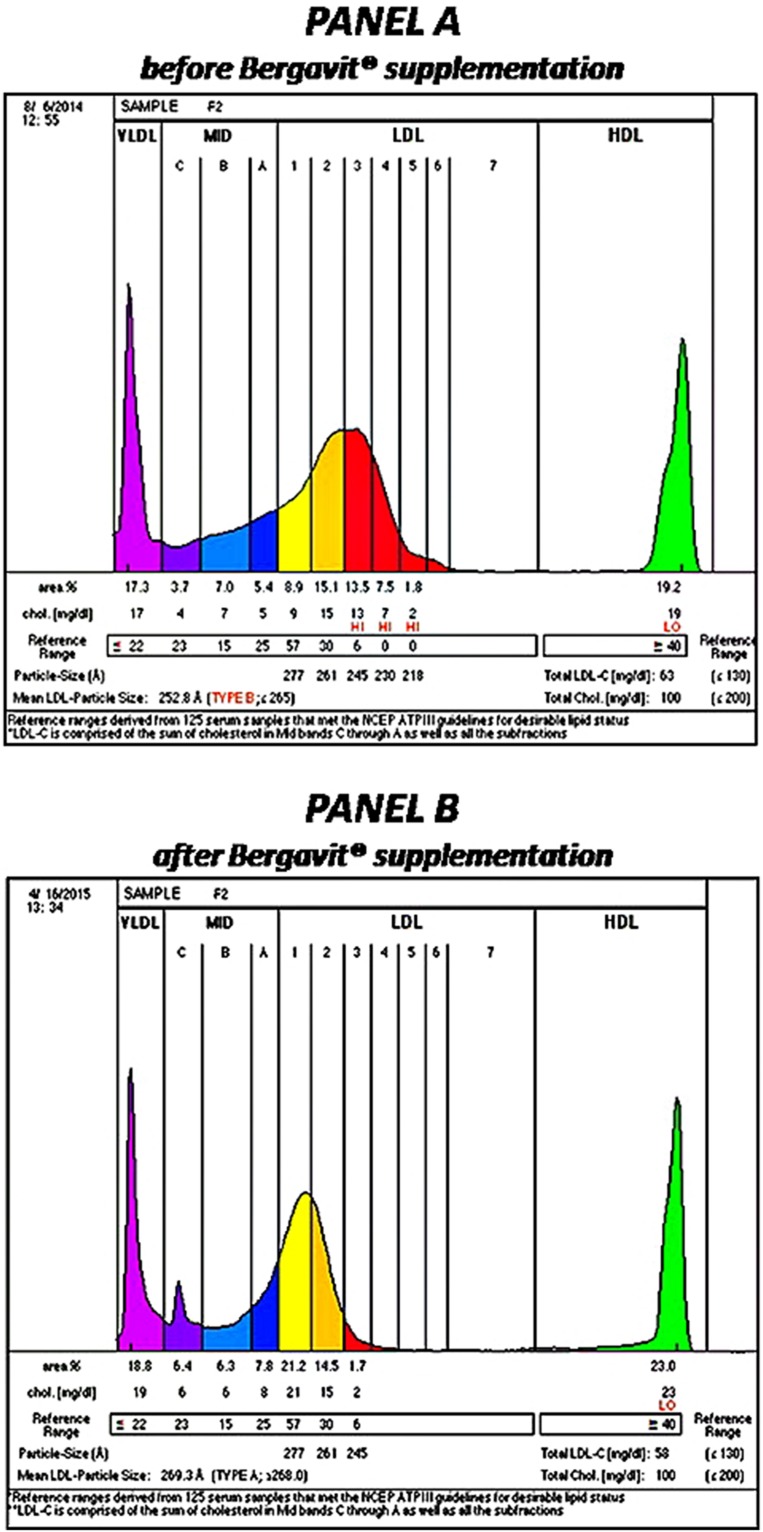
**Lipoprotein profile of a rapresentative subject before **(PANEL A)** and after **(PANEL B)** 6 months of Bergavit^®^ (150 mg of flavonoids, with 16% of neoeriocitrin, 47% of neohesperidin, and 37% of naringin, daily) supplementation.** Lipoprint color graphs are generated by lipoprint sytem using Lipoware Clinical Analysis Program.

After 6 months of Bergavit^®^ supplementation (data not shown) anthropometric parameters slightly improved (body weight, waist circumference, and body mass index), but the differences did not achieve the statistical significance. As shown in **Table 2**, plasma lipids significantly improved with a decrease in TC, LDL-C (*p* < 0.0001 for both) and TG (*p* = 0.0020), while HDL-C increased (*p* = 0.0007). Box plots of Δ LDL-C (%) based on quartiles of baseline plasma LDL-C levels are shown in **Figure 2**. A stronger reduction in plasma LDL-C level was achieved in subjects with higher baseline LDL-C levels (*p* = 0.004 for trend). In addition, large LDL1 particles increased by 20% (*p* < 0.0001), while small, dense LDL3, LDL4, and LDL5 decreased by 38, 53, and 67% (*p* < 0.0001, *p* = 0.0053, and *p* = 0.0133, respectively). This lead to a decreased LDL peak particle size (*p* < 0.0001). In addition (data not shown), large, medium, and small IDL significantly increased after 6 months of Bergavit^®^ supplementation, with the greatest increase in larger IDL-C subclasses, while no significant differences were found for VLDL particles. A representative lipoprotein profile for one subject before and after Bergavit^®^ supplementation is shown in **Figure 3**. Further, cIMT, as a marker of subclinical atherosclerosis, decreased after 6 months of Bergavit^®^ supplementation from 1.2 ± 0.4 to 0.9 ± 0.1 mm (25%; *p* < 0.0001; see **Table 2**).

**Table 2:** Effects of Bergavit^®^ (150 mg of flavonoids, with 16% of neoeriocitrin, 47% of neohesperidin, and 37% of naringin, daily) on plasma lipids, LDL subclasses and carotid intima-media thickness (IMT) in subjects with moderate hypercholesterolemia after 6 months of supplementation.

	Baseline	After 6 months	p	% Change
Total cholesterol (mmol/l) [mg/dl]	6.6 ± 0.4 [257 ± 15]	5.8 ± 1.1 [223 ± 41]	<0.0001	-12
HDL-cholesterol (mmol/l) [mg/dl]	1.3 ± 0.2 [48 ± 10]	1.4 ± 0.4 [52 ± 14]	0.0007	+8
Triglycerides (mmol/l) [mg/dl]	1.8 ± 0.6 [162 ± 54]	1.5 ± 0.9 [136 ± 79]	0.0020	-17
LDL-cholesterol (mmol/l) [mg/dl]	4.6 ± 0.2 [176 ± 8]	3.7 ± 1.0 [144 ± 37]	<0.0001	-20
Carotid IMT (mm)	1.2 ± 0.4	0.9 ± 0.1	<0.0001	-25
LDL size (angstrom)	264 ± 5	267 ± 7	<0.0001	+1
LDL-1 (%)	41.2 ± 0.2	49.6 ± 0.2	<0.0001	+20
LDL-2 (%)	40.8 ± 0.1	39.7 ± 0.2	0.4428	-3
LDL-3 (%)	14.5 ± 0.1	9.0 ± 0.1	<0.0001	-38
LDL-4 (%)	3.2 ± 0.1	1.5 ± 0.1	0.0053	-53
LDL-5 (%)	0.3 ± 0.0	0.1 ± 0.0	0.0133	-67
LDL-6 (%)	-	-	-	-
LDL-7 (%)	-	-	-	-


Finally, we performed a correlation analysis (data not shown) in order to assess potential associations between the observed changes in all evaluated parameters. A significant positive association was found between LDL-C and both TC (*r* = +0.908; *p* < 0.0001) and waist circumference (*r* = +0.273; *p* = 0.0143). Change in cIMT was associated with changes in the smallest LDL5 particles (*r* = +0.242; *p* = 0.031).

## Discussion

The findings of the present study indicate that Bergavit^®^ supplementation has beneficial effects on lipid levels including atherogenic LDL particles in subjects with moderate hypercholesterolemia. After 6 months of Bergavit^®^ supplementation lipid levels (TC, TG, and LDL-C) decreased, while the atherogenic lipid pattern improved (with increased presence of large and decreased presence of small, dense LDL particles). Our findings are somewhat consistent with previous studies (Mollace et al., 2011; Gliozzi et al., 2013, 2014). However, we have shown for the first time that supplementation with this extract reduced cIMT. This is of high clinical importance, since it is largely unknown how dietary supplements and nutraceuticals impact CV risk. The reduction in cIMT suggests that, consistent with its impact on LDL-C and small LDL subfractions, Bergavit^®^ may favorably impact CV risk in subjects with moderate hypercholesterolemia. Additional investigation will have to be performed in order to fully elucidate how the Bergavit^®^ components (Bergamot flavonoids) may regulate serum levels of atherogenic lipoproteins (Dugo et al., 2005; Nogata et al., 2006).

**FIGURE 4:**
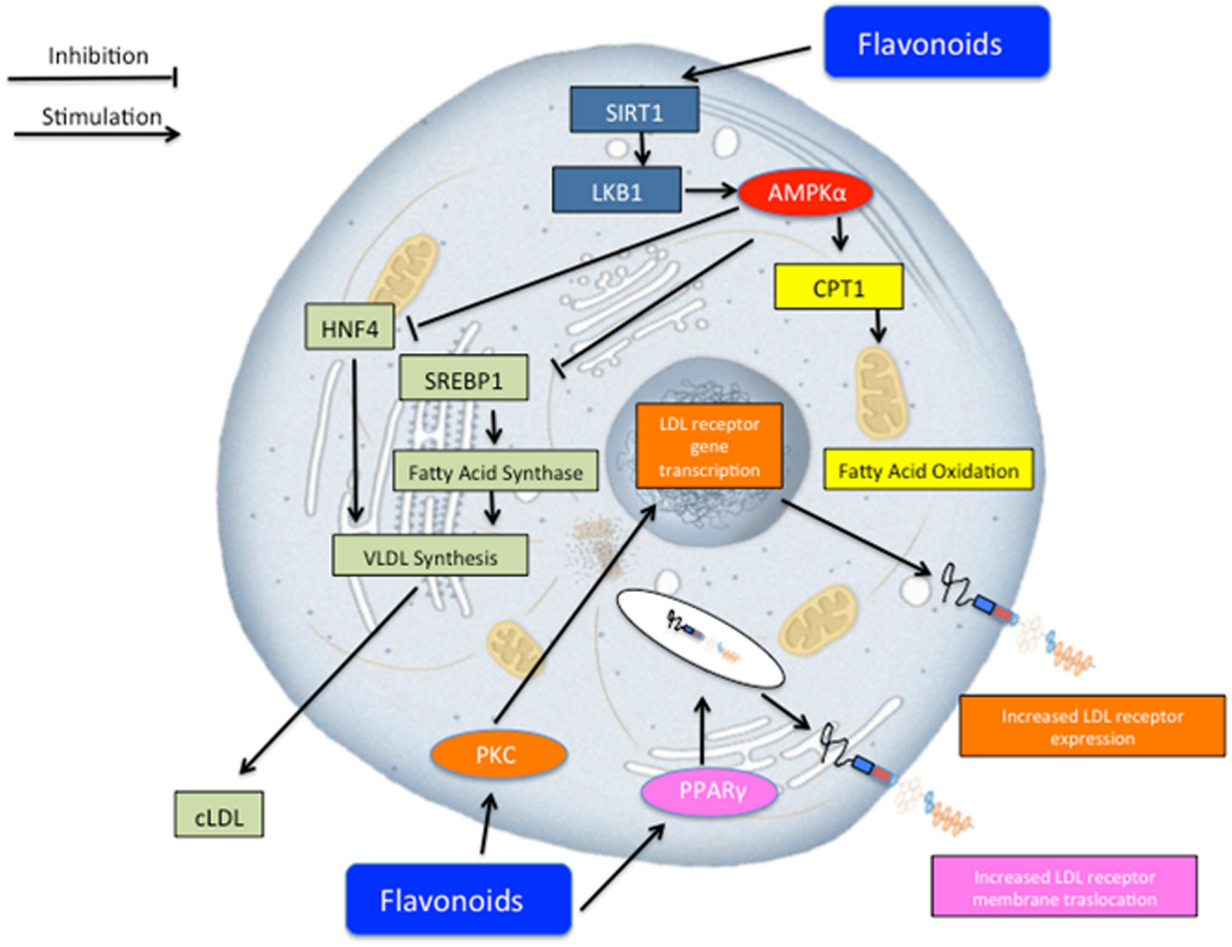
**Schematic representation of potential lipid lowering effect of Bergavit^®^ (Bergamot-derived flavonoids: 150 mg of flavonoids, with 16% of neoeriocitrin, 47% of neohesperidin and 37% of naringin, daily).** Several mechanisms may concur to regulate lipid homeostasis. Flavonoids may lead to an increase of lipid consumption via activation of mitochondria oxidation and at the same time flavonoids decrease very low-density lipoproteins (VLDL) synthesis. Other potential mechanisms include increased LDL receptor gene transcription via protein kinase C (PKC) activation and increased membrane translocation of this receptor via peroxisome proliferator-activated receptors gamma (PPAR-γ) activation. AMPK-α, adenosine monophosphate-activated protein kinase alpha; CPT1, carnitine palmitoyltransferase 1; HNF4, hepatocyte nuclear factor 4; SREBP-1, sterol regulatory element-binding protein 1.

A particular contribution to the lipid-lowering response appears to be due to neoeriocitrin, neohesperidin, and naringin, flavonoid glycosides present in Bergamot juice. Several mechanisms may be advocated in order to explain such results (**Figure 4**). Previous reports (Salamone et al., 2012; Andersen et al., 2014; Bruckbauer and Zemel, 2014; Dong et al., 2014; Tsutsumi et al., 2014) showed that flavonoids may activate sirtuin-1 which in turns activates adenosine monophosphate-activated protein kinase (AMPK)-α which may serve as a master switch regulator for cell metabolism. Such pharmacological effect, on one hand, leads to fatty acid oxidation via carnitine palmitoyltransferase 1 (CPT1) activation (Chang et al., 2013) and, on the other hand, reduces VLDL synthesis via the inhibition of hepatocyte nuclear factor 4 (HNF4; Reddy et al., 1999) and sterol regulatory element-binding protein 1 (SREBP-1; Quesada et al., 2009). Furthermore, other lipid lowering molecular mechanisms may include those related to the LDL receptor. To this regard, previous reports showed that flavonoids activates protein kinase C (PKC), which in turns triggers a series of biochemical events, leading to the transcription of the gene coding for LDL receptor (Kumar et al., 1997), and thus increasing its expression and concomitantly sequestering circulating LDL inside the cells. Finally, flavonoids may activate peroxisome proliferator-activated receptors (PPAR)-γ and thus trigger a series of molecular mechanisms leading to the translocation of LDL receptor on the plasma membrane for sequestering circulating LDL (Farras et al., 2013).

We found a significant modification in plasma lipoprotein subfractions after Bergavit^®^ supplementation that is somewhat consistent with our previous study where the effects of another nutraceutical (chitosan) was assessed in patients with hypertriglyceridemia [TG > 150 mg/dl (>3.9 mmol/l)] (Rizzo et al., 2014), as well as with other studies that used different nutraceuticals [as reviewed in (Patti et al., 2015)]. In the present study we found that small dense LDL particles (LDL3, -4, and -5) decreased, which may decrease CV risk (Mikhailidis et al., 2011a). In addition, IDL subfractions increased, while VLDL particles did not change significantly. IDL subclasses differ in their ability to predict CV risk, in the same way as it occurs for LDL subclasses (Srisawasdi et al., 2013). In this context, in the present study we found the greatest increase in larger IDL-C subclasses, which might be less atherogenic compared to the other subspecies.

Our results are somewhat consistent with findings from a study that included patients with metabolic syndrome where Bergamot-derived polyphenolic fraction was given before meals for 120 consecutive days (Gliozzi et al., 2014). In that study, LDL subfractions were evaluated by nuclear magnetic resonance spectroscopy, while we used a more detailed method. The effect of Bergavit^®^, observed in our study, may have important clinical significance because of its potential to influence the quality and not just the quantity of LDL-C. Small, dense LDL particles are indeed a recognized risk factor of CV risk, with strong atherogenic potential. Several mechanisms are involved in the enhanced atherogenicity of small dense LDL, including the increased filtration through the endothelium, a reduced LDL receptor affinity as well as a prolonged circulation time and higher proteoglycan binding; further, the oxidative modification of LDL is recognized as the key step in the atherothrombotic process, and small dense LDL exhibit enhanced oxidative susceptibility and lower levels of antioxidants (Rizzo and Berneis, 2006a,b; Rizzo et al., 2007; Nikolic et al., 2013; Garcia-Rios et al., 2014). In addition, in the last years a large number of studies, including epidemiologic studies as well as clinical intervention trials, have reported a strong association between CV risk and small, dense LDL; this topic has been reviewed and discussed by the European Panel of experts in a Consensus Statement on the pathophysiology, atherogenicity and clinical significance of LDL subclasses (Mikhailidis et al., 2011a). However, the mechanism by which the LDL particle shifts to a larger size cannot be fully discerned from these data. It is likely that the reductions in both TC and TG induced by Bergavit^®^ contribute to this phenomenon. Additionally, it has been shown that flavonoids may inhibit the oxidation of LDL as well as reduce oxidative stress *in vitro* (Fuhrman et al., 1995; Naderi et al., 2003).

It is widely accepted that elevated LDL-C levels are a major risk factor for coronary heart disease, and that excessive LDL in the blood is deposited in the blood vessel walls and becomes the major component of atherosclerosis plaque lesions (Rizzo et al., 2010). We found that Bergamot extract supplementation reduced LDL-C by 20% overall, with a stronger reduction seen in subjects with higher baseline plasma LDL-C levels. Yet, the novel finding of the present study was the beneficial effect on cIMT. In this context, changes in cIMT correlated with changes in the smallest LDL5 particles. This finding might have important clinical significance given that it has been shown that the smallest, most dense LDL particles are strong predictors of coronary disease risk (Williams et al., 2003). In addition, smaller peak LDL particle size predicts an increased risk for myocardial infarction (Stampfer et al., 1996; Austin et al., 1988) and proposed mechanisms seem to be related to a greater atherogenicity of smaller, denser LDL compared to larger, more buoyant LDL particles (Mikhailidis et al., 2011a,b).

Among dietary supplements it has been shown that polyphenols can exert beneficial effects on vascular and CVD protection, such as antioxidant, anti-platelet, anti-inflammatory effects, and may improve endothelial function and contribute to stabilization of atheromatous plaque (Pandey and Rizvi, 2009). However, more randomized clinical trial data establishing nutraceuticals’ efficacy for reducing CV risk are needed. For instance, soy and red yeast rice seem to have effects on CV events/mortality (Mannarino et al., 2014; Lu et al., 2008), while some nutraceuticals seem to beneficially affect surrogate markers of vascular damage, such as arterial IMT, endothelial dysfunction and arterial stiffness (Houston et al., 2009). Results obtained in animal models support the hypolipemic and vasoprotective effects of Bergamot constituents such as various bioflavonoids (Choe et al., 2001; Mollace et al., 2008), indicating that *Citrus* flavonoids might prevent atherosclerosis (Yu et al., 2005; Miceli et al., 2007).

Potential limitations of the present study include the absence of a control (placebo) group, while its strengths include full treatment adherence as well as the blinded measurements of biochemical parameters, lipoprotein subclasses, as well as a blinded manner in assessing cIMT. We used high-quality methodology to assess the full spectrum of LDL subclasses and for the first time demonstrated beneficial effects on subclinical atherosclerosis.

## Conclusion

This is the first study investigating the effect of Bergavit^®^ supplementation on cardio-metabolic risk in dyslipidemic subjects. Bergavit^®^ supplementation significantly lowered plasma lipids (TC, TG, and LDL-C) and improved the lipoprotein profile by decreasing atherogenic small, dense LDL particles. Of great clinical significance is that Bergavit^®^ significantly reduced cIMT after 6 months of supplementation. Consequently, Bergavit^®^ may represent a safe, alternative therapeutic approach, especially in subjects suffering from statin intolerance, that may contribute to a diminished risk for atherosclerosis. These findings shed a new light on the potential use of Bergamot-extract supplements in the prevention and/or reduction of overall cardiometabolic risk.

## Author Contributions

MR, GM, and DN designed the study. AMP, RVG, and DN researched data and wrote the manuscript. TB, FG, and SD researched data. PT, AR, MR, and GM contributed to the discussion and reviewed/edited the manuscript. The guarantor for this work is MR. All authors approved the final manuscript.

## Conflict of Interest Statement

The authors declare that the research was conducted in the absence of any commercial or financial relationships that could be construed as a potential conflict of interest.

The authors did not receive financial or professional help with the preparation of the manuscript. The authors have given talks, attended conferences and participated in advisory boards and trials sponsored by various pharmaceutical companies.

## References

[B1] Adhyaru B. B. Jacobson T. A. (2015). New cholesterol guidelines for the management of atherosclerotic cardiovascular disease risk: a comparison of the 2013 American College of Cardiology/American Heart Association cholesterol guidelines with the 2014 National Lipid Association recommendations for patient-centered management of dyslipidemia. *Cardiol. Clin.* 33 181–196. 10.1016/j.ccl.2015.02.001 25939292

[B2] Alagona P. Jr. Ahmad T. A. (2015). Cardiovascular disease risk assessment and prevention: current guidelines and limitations. *Med. Clin. North Am.* 99 711–731. 10.1016/j.mcna.2015.02.003 26042878

[B3] Andersen C. Kotowska D. Tortzen C. G. Kristiansen K. Nielsen J. Petersen R. K. (2014). 2-(2-Bromophenyl)-formononetin and 2-heptyl-formononetin are PPARgamma partial agonists and reduce lipid accumulation in 3T3-L1 adipocytes. *Bioorg. Med. Chem.* 22 6105–6111. 10.1016/j.bmc.2014.08.037 25262940

[B4] Austin M. A. Breslow J. L. Hennekens C. H. Buring J. E. Willett W. C. Krauss R. M. (1988). Low-density lipoprotein subclass patterns and risk of myocardial infarction. *JAMA* 260 1917–1921. 10.1001/jama.1988.03410130125037 3418853

[B5] Banach M. Rizzo M. Toth P. P. Farnier M. Davidson M. H. Al-Rasadi K. (2015a). Statin intolerance - an attempt at a unified definition. Position paper from an International Lipid Expert Panel. *Expert Opin. Drug Saf.* 14 935–955. 10.1517/14740338.2015.1039980 25907232

[B6] Banach M. Rizzo M. Toth P. P. Farnier M. Davidson M. H. Al-Rasadi K. (2015b). Statin intolerance - an attempt at a unified definition. Position paper from an International Lipid Expert Panel. *Arch. Med. Sci.* 11 1–23. 10.5114/aoms.2015.49807 25861286 PMC4379380

[B7] Benavente-Garcia O. Castillo J. (2008). Update on uses and properties of citrus flavonoids: new findings in anticancer, cardiovascular, and anti-inflammatory activity. *J. Agric. Food Chem.* 56 6185–6205. 10.1021/jf8006568 18593176

[B8] Berneis K. Rizzo M. Berthold H. K. Spinas G. A. Krone W. Gouni-Berthold I. (2010). Ezetimibe alone or in combination with simvastatin increases small dense low-density lipoproteins in healthy men: a randomized trial. *Eur. Heart J.* 31 1633–1639. 10.1093/eurheartj/ehq181 20525999

[B9] Bruckbauer A. Zemel M. B. (2014). Synergistic effects of polyphenols and methylxanthines with Leucine on AMPK/Sirtuin-mediated metabolism in muscle cells and adipocytes. *PLoS ONE* 9:e89166 10.1371/journal.pone.0089166 PMC3925247 24551237

[B10] Chang J. J. Hsu M. J. Huang H. P. Chung D. J. Chang Y. C. Wang C. J. (2013). Mulberry anthocyanins inhibit oleic acid induced lipid accumulation by reduction of lipogenesis and promotion of hepatic lipid clearance. *J. Agric. Food Chem.* 61 6069–6076. 10.1021/jf401171k 23731091

[B11] Choe S. C. Kim H. S. Jeong T. S. Bok S. H. Park Y. B. (2001). Naringin has an antiatherogenic effect with the inhibition of intercellular adhesion molecule-1 in hypercholesterolemic rabbits. *J. Cardiovasc. Pharmacol.* 38 947–955. 10.1097/00005344-200112000-00017 11707699

[B12] Corrado E. Rizzo M. Tantillo R. Muratori I. Bonura F. Vitale G. (2006). Markers of inflammation and infection influence the outcome of patients with baseline asymptomatic carotid lesions: a 5-year follow-up study. *Stroke* 37 482–486. 10.1161/01.STR.0000198813.56398.14 16373649

[B13] Di Donna L. Iacopetta D. Cappello A. R. Gallucci G. Martello E. Fiorillo M. (2014). Hypocholesterolaemic activity of 3-hydroxy-3-methyl-glutaryl flavanones enriched fraction from bergamot fruit (*Citrus bergamia*): “In vivo” studies. *J. Funct. Foods* 7 558–568. 10.1016/j.jff.2013.12.029

[B14] Dong J. Zhang X. Zhang L. Bian H. X. Xu N. Bao B. (2014). Quercetin reduces obesity-associated ATM infiltration and inflammation in mice: a mechanism including AMPKalpha1/SIRT1. *J. Lipid Res.* 55 363–374. 10.1194/jlr.M038786 24465016 PMC3934722

[B15] Dugo P. Presti M. L. Ohman M. Fazio A. Dugo G. Mondello L. (2005). Determination of flavonoids in citrus juices by micro-HPLC-ESI/MS. *J. Sep. Sci.* 28 1149–1156. 10.1002/jssc.200500053 16116991

[B16] Farras M. Valls R. M. Fernandez-Castillejo S. Giralt M. Sola R. Subirana I. (2013). Olive oil polyphenols enhance the expression of cholesterol eﬄux related genes in vivo in humans. A randomized controlled trial. *J. Nutr. Biochem.* 24 1334–1339. 10.1016/j.jnutbio.2012.10.008 23333095

[B17] Fuhrman B. Lavy A. Aviram M. (1995). Consumption of red wine with meals reduces the susceptibility of human plasma and low-density lipoprotein to lipid peroxidation. *Am. J. Clin. Nutr.* 61 549–554. 7872219 10.1093/ajcn/61.3.549

[B18] Garcia-Rios A. Nikolic D. Perez-Martinez P. Lopez-Miranda J. Rizzo M. Hoogeveen R. C. (2014). LDL and HDL subfractions, dysfunctional HDL: treatment options. *Curr. Pharm. Des.* 20 6249–6255. 10.2174/1381612820666140620154014 24953394

[B19] Gliozzi M. Carresi C. Musolino V. Palma E. Muscoli C. Vitale C. (2014). The effect of bergamot-derived polyphenolic fraction on ldl small dense particles and non alcoholic fatty liver disease in patients with metabolic syndrome. *Adv. Biol. Chem.* 4 129–137. 10.4236/abc.2014.42017

[B20] Gliozzi M. Walker R. Muscoli S. Vitale C. Gratteri S. Carresi C. (2013). Bergamot polyphenolic fraction enhances rosuvastatin-induced effect on LDL-cholesterol, LOX-1 expression and protein kinase B phosphorylation in patients with hyperlipidemia. *Int. J. Cardiol.* 170 140–145. 10.1016/j.ijcard.2013.08.125 24239156

[B21] Graziano A. C. Cardile V. Crasci L. Caggia S. Dugo P. Bonina F. (2012). Protective effects of an extract from *Citrus bergamia* against inflammatory injury in interferon-gamma and histamine exposed human keratinocytes. *Life Sci.* 90 968–974. 10.1016/j.lfs.2012.04.043 22634580

[B22] Houston M. C. Fazio S. Chilton F. H. Wise D. E. Jones K. B. Barringer T. A. (2009). Nonpharmacologic treatment of dyslipidemia. *Prog. Cardiovasc. Dis.* 52 61–94. 10.1016/j.pcad.2009.02.002 19732602

[B23] Kumar A. Chambers T. C. Cloud-Heflin B. A. Mehta K. D. (1997). Phorbol ester-induced low density lipoprotein receptor gene expression in HepG2 cells involves protein kinase C-mediated p42/44 MAP kinase activation. *J. Lipid Res.* 38 2240–2248. 9392422

[B24] Lu Z. Kou W. Du B. Wu Y. Zhao S. Brusco O. A. (2008). Effect of Xuezhikang, an extract from red yeast Chinese rice, on coronary events in a Chinese population with previous myocardial infarction. *Am. J. Cardiol.* 101 1689–1693. 10.1016/j.amjcard.2008.02.056 18549841

[B25] Mannarino M. R. Ministrini S. Pirro M. (2014). Nutraceuticals for the treatment of hypercholesterolemia. *Eur. J. Int. Med.* 25 592–599. 10.1016/j.ejim.2014.06.008 24997485

[B26] Miceli N. Mondello M. R. Monforte M. T. Sdrafkakis V. Dugo P. Crupi M. L. (2007). Hypolipidemic effects of *Citrus bergamia* Risso et Poiteau juice in rats fed a hypercholesterolemic diet. *J. Agric. Food Chem.* 55 10671–10677. 10.1021/jf071772i 18038978

[B27] Mikhailidis D. P. Elisaf M. Rizzo M. Berneis K. Griffin B. Zambon A. (2011a). “European panel on low density lipoprotein (LDL) subclasses:” a statement on the pathophysiology, atherogenicity and clinical significance of LDL subclasses. *Curr. Vasc. Pharmacol.* 9 533–571. 10.2174/157016111796642698 21595628

[B28] Mikhailidis D. P. Elisaf M. Rizzo M. Berneis K. Griffin B. Zambon A. (2011b). “European panel on low density lipoprotein (LDL) subclasses:” a statement on the pathophysiology, atherogenicity and clinical significance of LDL subclasses: executive summary. *Curr. Vasc. Pharmacol.* 9 531–532. 10.2174/157016111796642661 21595629

[B29] Mollace V. Ragusa S. Sacco I. Muscoli C. Sculco F. Visalli V. (2008). The protective effect of bergamot oil extract on lecitine-like oxyLDL receptor-1 expression in balloon injury-related neointima formation. *J. Cardiovasc. Pharmacol. Ther.* 13 120–129. 10.1177/1074248407313821 18413898

[B30] Mollace V. Sacco I. Janda E. Malara C. Ventrice D. Colica C. (2011). Hypolipemic and hypoglycaemic activity of bergamot polyphenols: from animal models to human studies. *Fitoterapia* 82 309–316. 10.1016/j.fitote.2010.10.014 21056640

[B31] Naderi G. A. Asgary S. Sarraf-Zadegan N. Shirvany H. (2003). Anti-oxidant effect of flavonoids on the susceptibility of LDL oxidation. *Mol. Cell. Biochem.* 246 193–196. 10.1023/A:1023483223842 12841362

[B32] Nikolic D. Katsiki N. Montalto G. Isenovic E. R. Mikhailidis D. P. Rizzo M. (2013). Lipoprotein subfractions in metabolic syndrome and obesity: clinical significance and therapeutic approaches. *Nutrients* 5 928–948. 10.3390/nu5030928 23507795 PMC3705327

[B33] Nogata Y. Sakamoto K. Shiratsuchi H. Ishii T. Yano M. Ohta H. (2006). Flavonoid composition of fruit tissues of citrus species. *Biosci. Biotechnol. Biochem.* 70 178–192. 10.1271/bbb.70.178 16428836

[B34] Pandey K. B. Rizvi S. I. (2009). Plant polyphenols as dietary antioxidants in human health and disease. *Oxid Med. Cell Longev.* 2 270–278. 10.4161/oxim.2.5.9498 20716914 PMC2835915

[B35] Patti A. M. Katsiki N. Nikolic D. Al-Rasadi K. Rizzo M. (2015). Nutraceuticals in lipid-lowering treatment: a narrative review on the role of chitosan. *Angiology* 66 416–421. 10.1177/0003319714542999 25037700

[B36] Quesada H. del Bas J. M. Pajuelo D. Diaz S. Fernandez-Larrea J. Pinent M. (2009). Grape seed proanthocyanidins correct dyslipidemia associated with a high-fat diet in rats and repress genes controlling lipogenesis and VLDL assembling in liver. *Int. J. Obes (Lond.)* 33 1007–1012. 10.1038/ijo.2009.136 19581912

[B37] Reddy S. Yang W. Taylor D. G. Shen X. Oxender D. Kust G. (1999). Mitogen-activated protein kinase regulates transcription of the ApoCIII gene. Involvement of the orphan nuclear receptor HNF4. *J. Biol. Chem.* 274 33050–33056. 10.1074/jbc.274.46.33050 10551874

[B38] Risitano R. Curro M. Cirmi S. Ferlazzo N. Campiglia P. Caccamo D. (2014). Flavonoid fraction of Bergamot juice reduces LPS-induced inflammatory response through SIRT1-mediated NF-kappaB inhibition in THP-1 monocytes. *PLoS ONE* 9:e107431 10.1371/journal.pone.0107431 PMC4178028 25260046

[B39] Rizzo M. Berneis K. (2006a). Effect of statins on low-density lipoprotein size: a new role in cardiovascular prevention? *South Med. J.* 99 1015–1016. 10.1097/01.smj.0000235471.56184.77 17004543

[B40] Rizzo M. Berneis K. (2006b). The clinical relevance of low-density-lipoproteins size modulation by statins. *Cardiovasc. Drugs. Ther.* 20 205–217. 10.1007/s10557-006-8283-x 16775666

[B41] Rizzo M. Berneis K. Koulouris S. Pastromas S. Rini G. B. Sakellariou D. (2010). Should we measure routinely oxidised and atherogenic dense low-density lipoproteins in subjects with type 2 diabetes? *Int. J. Clin. Pract.* 64 1632–1642. 10.1111/j.1742-1241.2010.02378.x 20831734

[B42] Rizzo M. Giglio R. V. Nikolic D. Patti A. M. Campanella C. Cocchi M. (2014). Effects of chitosan on plasma lipids and lipoproteins: a 4-month prospective pilot study. *Angiology* 65 538–542. 10.1177/0003319713493126 23785043

[B43] Rizzo M. Rini G. B. Berneis K. (2007). Effects of statins, fibrates, rosuvastatin, and ezetimibe beyond cholesterol: the modulation of LDL size and subclasses in high-risk patients. *Adv. Ther.* 24 575–582. 10.1007/BF02848780 17660166

[B44] Sakurada T. Mizoguchi H. Kuwahata H. Katsuyama S. Komatsu T. Morrone L. A. (2011). Intraplantar injection of bergamot essential oil induces peripheral antinociception mediated by opioid mechanism. *Pharmacol. Biochem. Behav.* 97 436–443. 10.1016/j.pbb.2010.09.020 20932858

[B45] Salamone F. Galvano F. Cappello F. Mangiameli A. Barbagallo I. Li Volti G. (2012). Silibinin modulates lipid homeostasis and inhibits nuclear factor kappa B activation in experimental nonalcoholic steatohepatitis. *Transl. Res.* 159 477–486. 10.1016/j.trsl.2011.12.003 22633099

[B46] Scichilone N. Rizzo M. Benfante A. Catania R. Giglio R. V. Nikolic D. (2013). Serum low density lipoprotein subclasses in asthma. *Respir. Med.* 107 1866–1872. 10.1016/j.rmed.2013.09.001 24075885

[B47] Srisawasdi P. Vanavanan S. Rochanawutanon M. Pornsuriyasak P. Tantrakul V. Kruthkul K. (2013). Heterogeneous properties of intermediate- and low-density lipoprotein subpopulations. *Clin. Biochem.* 46 1509–1515. 10.1016/j.clinbiochem.2013.06.021 23830843

[B48] Stampfer M. J. Krauss R. M. Ma J. Blanche P. J. Holl L. G. Sacks F. M. (1996). A prospective study of triglyceride level, low-density lipoprotein particle diameter, and risk of myocardial infarction. *JAMA* 276 882–888. 10.1001/jama.276.11.882 8782637

[B49] Trombetta D. Cimino F. Cristani M. Mandalari G. Saija A. Ginestra G. (2010). In vitro protective effects of two extracts from bergamot peels on human endothelial cells exposed to tumor necrosis factor-alpha (TNF-alpha). *J. Agric. Food Chem.* 58 8430–8436. 10.1021/jf1008605 20578719

[B50] Tsutsumi R. Yoshida T. Nii Y. Okahisa N. Iwata S. Tsukayama M. (2014). Sudachitin, a polymethoxylated flavone, improves glucose and lipid metabolism by increasing mitochondrial biogenesis in skeletal muscle. *Nutr. Metab. (Lond.)* 11:32 10.1186/1743-7075-11-32 PMC4128574 25114710

[B51] Williams P. T. Superko H. R. Haskell W. L. Alderman E. L. Blanche P. J. Holl L. G. (2003). Smallest LDL particles are most strongly related to coronary disease progression in men. *Arterioscler. Thromb. Vasc. Biol.* 23 314–321. 10.1161/01.ATV.0000053385.64132.2D 12588777

[B52] Yu J. Wang L. Walzem R. L. Miller E. G. Pike L. M. Patil B. S. (2005). Antioxidant activity of citrus limonoids, flavonoids, and coumarins. *J. Agric. Food Chem.* 53 2009–2014. 10.1021/jf0484632 15769128

